# MICy: a Novel Flow Cytometric Method for Rapid Determination of Minimal Inhibitory Concentration

**DOI:** 10.1128/spectrum.00901-21

**Published:** 2021-12-08

**Authors:** András Kállai, Márta Kelemen, Noémi Molnár, Adrienn Tropotei, Balázs Hauser, Zsolt Iványi, János Gál, Erzsébet Ligeti, Katalin Kristóf, Ákos M. Lőrincz

**Affiliations:** a Department of Anaesthesiology and Intensive Therapy, Semmelweis University, Budapest, Hungary; b Department of Physiology, Semmelweis University, Budapest, Hungary; c Clinical Microbiology Laboratory, Department of Laboratory Medicine, Semmelweis University, Budapest, Hungary; d Second Department of Internal Medicine, St George’s Hospital, Székesfehérvár, Hungary; Hartford Hospital

**Keywords:** flow cytometry, MIC, antibiotic susceptibility testing, rapid tests

## Abstract

Early initiated adequate antibiotic treatment is essential in intensive care. Shortening the length of antibiotic susceptibility testing (AST) can accelerate clinical decision-making. Our objective was to develop a simple flow cytometry (FC)-based AST that produces reliable results within a few hours. We developed a FC-based AST protocol (MICy) and tested it on six different bacteria strains (Escherichia coli, Klebsiella pneumoniae, Pseudomonas aeruginosa, Staphylococcus aureus, Streptococcus pyogenes, Enterococcus faecalis) in Mueller-Hinton and Luria–Bertani broth. We monitored the bacterial growth by FC to define the optimal time of AST. All bacteria were tested against 12 antibiotics and the MIC values were compared to microdilution used as reference method. McNemar and Fleiss’ kappa inter-observer tests were performed to analyze the bias between the two methods. Susceptibility profiles of the two methods were also compared. We found that FC is able to detect the bacterial growth after 4-h incubation. The point-by-point comparison of MICy and microdilution resulted in exact match above 87% (2642/3024) of all measurements. The MIC values obtained by MICy and microdilution agreed over 80% (173/216) within ±1 dilution range that gives a substantial inter-observer agreement with weighted Fleiss’ kappa. By using the EUCAST clinical breakpoints, we defined susceptibility profiles of MICy that were identical to microdilution in more than 92% (197/213) of the decisions. MICy resulted 8.7% major and 3.2% very major discrepancies. MICy is a new, simple FC-based AST method that produces susceptibility profile with low failure rate a workday earlier than the microdilution method.

**IMPORTANCE** MICy is a new, simple and rapid flow cytometry based antibiotic susceptibility testing (AST) method that produces susceptibility profile a workday earlier than the microdilution method or other classical phenotypic AST methods. Shortening the length of AST can accelerate clinical decision-making as targeted antibiotic treatment improves clinical outcomes and reduces mortality, duration of artificial ventilation, and length of stay in intensive care unit. It can also reduce nursing time and costs and the spreading of antibiotic resistance. In this study, we present the workflow and methodology of MICy and compare the results produced by MICy to microdilution step by step.

## INTRODUCTION

Proper antibiotic treatment is essential in all disciplines of medicine, especially in intensive care. Targeted antibiotic treatment based on antimicrobial susceptibility testing (AST) reduces mortality, duration of artificial ventilation, and length of stay in intensive care unit (1–3). It can also reduce nursing time and costs (4) and the spreading of antibiotic resistance (5, 6). Up-to-date clinical guidelines identified early AST as one of the most critical issues that need to be improved (5, 7, 8). Numerous studies report methods that can determine the susceptibility profile of a pathogen faster (9). An ideal AST should be reliable, fast, inexpensive, automatized, capable of high throughput, and coupled with simple data processing (10, 11). Still, the most frequently used diagnostic methods in clinical practice are the classical techniques such as microdilution. These methods are simple and capable of high throughput but also slow and labor intensive (9, 12). Although several new methods have been developed following the suggestions of the WHO (13), no test became superior to the classical methods in clinical practice (9, 12).

Flow cytometry (FC) is a robust technique that is able to detect bacteria as single particles, and it can also provide information on the integrity and the viability of antibiotic-treated bacteria (14–16). Despite many advantages of FC, it is still not implemented in the practice of clinical microbiology. The hitherto presented FC based AST studies focused on changes in light scattering characteristics (17, 18), membrane potential (19, 20) and membrane permeability (21, 22) to differentiate dead and viable bacteria. Although these parameters are crucial indicators of bacterial viability, there are no existing clinical standards or reference values to interpret changes of these parameters. No systematic clinical studies were performed to validate these preliminary FC observations. Moreover, the FC assays are challenging and need complex data processing and experts for data evaluation (23). These requirements and the lack of clinical experience are strong limitations of the use of multi-parametric FC-based non-phenotypic ASTs (12, 24). Some studies applied FC bacteria counting to follow the bacterial growth after antibiotic treatment (25, 26). Although these methods resulted in phenotypic MIC values, no systematic study was performed to validate these methods. Accordingly, FC based AST has reached limited success and the early scientific interest declined in the last 10 years (9, 12).

In a recent study, our group demonstrated that an FC-based assay can reliably quantify the antibacterial effect of neutrophils and subcellular structures a workday earlier than the reference methods (27). Based on these observations, we hypothesized that FC is suitable for rapid AST as well. In this study, we present a simple FC-based AST named “MICy” (combined from MIC and cytometry) that measures bacterial count changes in the presence of antibiotics. We compared the measured phenotypic MIC values and the defined susceptibility profiles to the gold standard.

## RESULTS

### Fluorescent labeling and fixation of bacteria.

**FIG 1:**
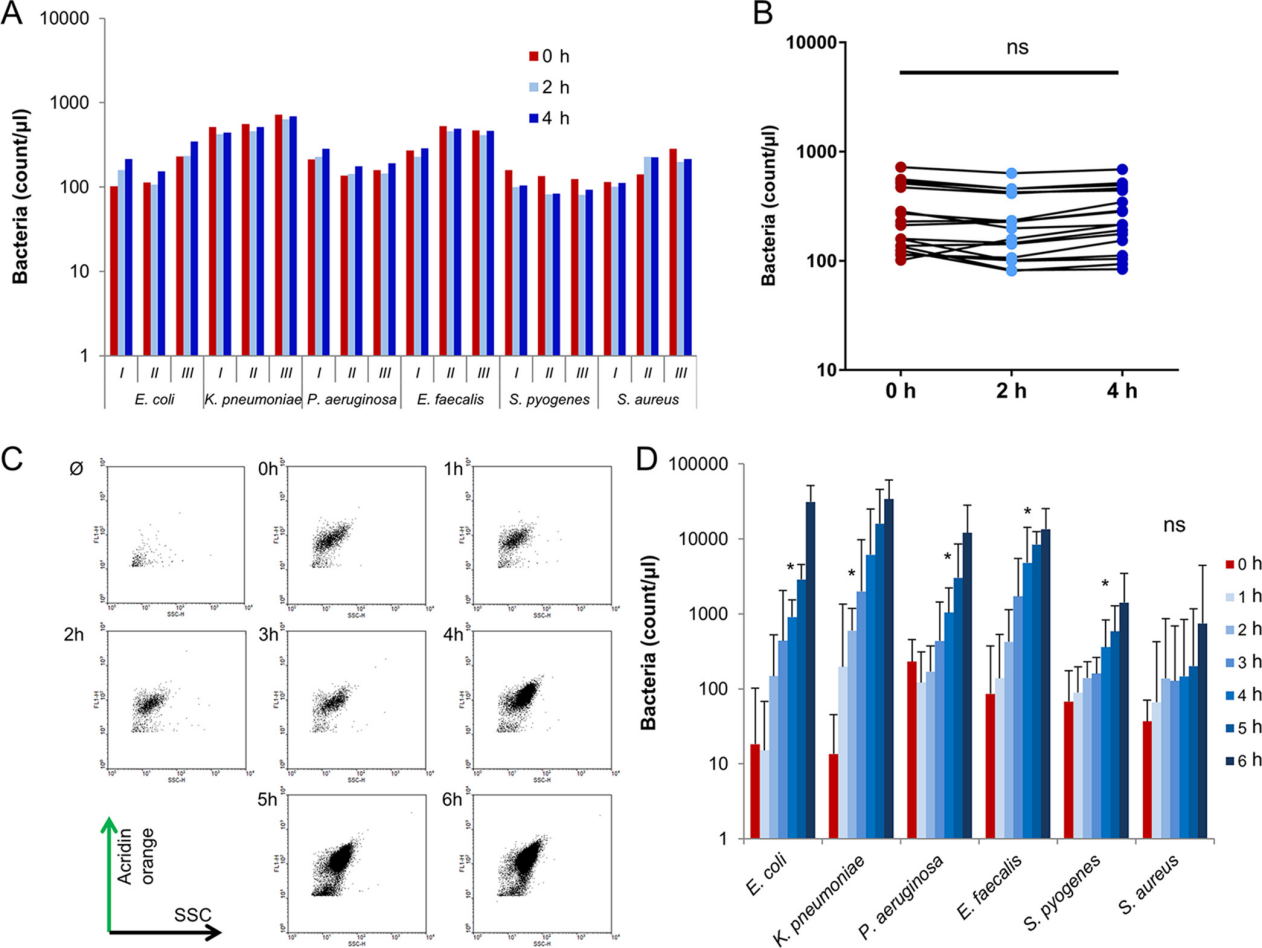
Determination of incubation time for detectable bacterial growth and stability of fluorescent labeling. (A) FC quantification of AO labeled bacteria immediately after fixation and 5 min labeling (red bars) and 2 (light blue bars) or 4 (dark blue bars) hours later. Roman numbers indicate three independent measurements. (B) Scatter diagram of all the 18 measurements of panel A. Data were analyzed by one-way RM ANOVA analysis with Tukey’s *post hoc* test. (C) Representative dot plots of *E.coli* samples tested at the indicated length of incubation. Ø represents the non-inoculated MH broth. (D) FC quantification of the change of bacterial count to monitor bacterial growth. Samples were taken in every hour up to 6 h. Mean + SEM, *n* = 3. Data were analyzed with one-way RM ANOVA with Dunetts’s multiple-comparison test. *, *P* < 0.05.

Labeling the bacteria and stopping their growth is a reasonable one-step process. Fig. 1A demonstrates three independent measurements of Gram positive and negative bacteria analyzed by FC immediately after fixation or 2 and 4 h later. During the test period, samples were held at room temperature under usual laboratory light exposition. Neither significant bacterial growth nor detectable decrease of the number of fluorescent particles (due to fluorescence quenching) was observed (Fig. 1B).

### Determination of incubation time for reliably detectable bacterial growth.

We inoculated six different bacterial species into MH and LB broth and measured the changes of bacterial count to determine the sufficient time for FC monitoring of bacterial growth. Some bacteria showed no clearly detectable growth in the first 3 h of incubation either in LB (not shown) or in MH (Fig. 1C and D). After a 4 h incubation, bacterial count began to increase continuously in the case of all tested bacteria (Fig. 1D) except the aggregate-forming S. aureus (see later). Since prior studies reported promising data after 2 h incubation (22, 26), we performed pilot measurements with 2 h incubation, but these measurements resulted in poor quality data as bacteria did not reach log phase (not shown). In the following experiments, we applied 4 h incubation for MICy measurements.

### Empirical definition of flow cytometric MIC value.

We performed 432 AST measurements both with microdilution and MICy: six species of bacteria were tested against 12 antibiotics in three independent repeats both in LB and MH broth. After pairing the FC data with microdilution results, we empirically defined two rules to convert the parametric data of MICy into binary results (growth or inhibition). The first rule refers to bacterial growth: MICy measurements may not be evaluated if the growth rate was lower than 4-fold during the incubation period. Growth rate was measured as the ratio of positive control and the initial bacterial count.

$$N_{\mathrm{positive}\;\mathrm{control}/}N_{\mathrm{initial}}\;\geq \;4$$

In the case of lower growth ratio, the FC counting was not precise enough to differentiate between growth and inhibition.

The second rule was set to define a cut-off value that discriminates “grown up” samples from inhibited samples. The empirically defined cut-off value was equal to the initial bacterial count. In cases when MICy measured no increased bacteria count than the initial bacterial count, we found inhibition with microdilution a day later. However, microdilution indicated inhibition also in some cases when bacterial count exceeded the initial bacterial count, but there was no further increase in the following dilution. Combining these two observations, we defined the second rule: The first “grown up” sample in an antibiotic serial dilution is the point where the bacterial count exceeds the initial bacterial count and the following serial dilution point exceeds the double of the initial count. Regarding the lowest tested antibiotic concentration (where there was no following data point), we defined it as “grown up” if it was above the initial bacterial count.

After converting the FC data into binary (growth and inhibition) results, MIC was defined as usual: the lowest concentration of an antibiotic that inhibited the bacterial growth.

### Modified evaluation of staphylococcal samples.

During the analysis of S. aureus samples, we became aware of an intriguing phenomenon. During the testing, staphylococci start to form aggregates. These aggregates disturb FC counting, since the same amount of bacteria appears as fewer but larger events. This results in a decrease in density of the bacterial population and shifts its geometric mean to higher SSC values (Fig. S2A). To avoid underestimation of bacterial growth, we measured both the event number and the geometric mean of SSC and multiplied them to get a combined parameter. This combined parameter was compared to the similarly generated parameter of the initial bacterial sample by using the second rule.

### Point-by-point comparison of data generated by microdilution and MICy.

To reveal the possible bias of the FC measurement, we compared the results of all data points produced by MICy to the parallel microdilution. We found exact match in more than 87% of all measurements regardless of the used medium. The inter-observer agreements Fleiss’ kappa showed substantial agreement between MICy and the microdilution (Table 1 and Table S1). Although there was a better match in case of Gram negative than in the case of Gram positive bacteria, the percentage of +/− (reference/MICy) mismatch was higher in the Gram negative group. The summarized +/− mismatch percentage was around 3% in both MH and LB (Table 1). On the other hand, the −/+ mismatch rate was higher than +/− mismatch rate (Table 1). The unequal distribution of errors was confirmed by the McNemar test (*P* < 0.0001).

**TABLE 1:** Summary table of point-by-point comparison of data generated by microdilution and MICy^*a*^

Broth	Bacteria	Measurement	+/+	−/−	Match %	−/+	+/−	Mismatch %	Fleiss’ kappa ± SE
Both	All	3024	1271	1371	87.4%	286 (9.6%)	96 (3.2%)	12.6%	0.748 ± 0.012
LB	Gram positive	756	234	413	85.6%	103 (13.6%)	6 (0.8%)	14.4%	0.700 ± 0.026
	Gram negative	756	394	277	88.8%	36 (4.8%)	49 (6.5%)	11.2%	0.770 ± 0.024
LB sum	Both	1512	628	690	87.2%	139 (9.2%)	55 (3.6%)	12.8%	0.744 ± 0.017
MH	Gram positive	756	249	397	85.4%	98 (12.9%)	12 (1.6%)	14.6%	0.701 ± 0.026
	Gram negative	756	394	284	89.7%	49 (6.5%)	29 (3.8%)	10.3%	0.789 ± 0.023
MH sum	Both	1512	643	681	87.6%	147 (9.7%)	41 (2.7%)	12.4%	0.752 ± 0.017

a Indicated numbers represent the number of data points fit in the column. Percentages represent the ratio to all measurements in the category. “+” represents grown up sample, “−” represents inhibition. First part of relations (before slash) refers to microdilution, second part (after slash) to MICy.

### Comparison of MIC values.

**FIG 2:**
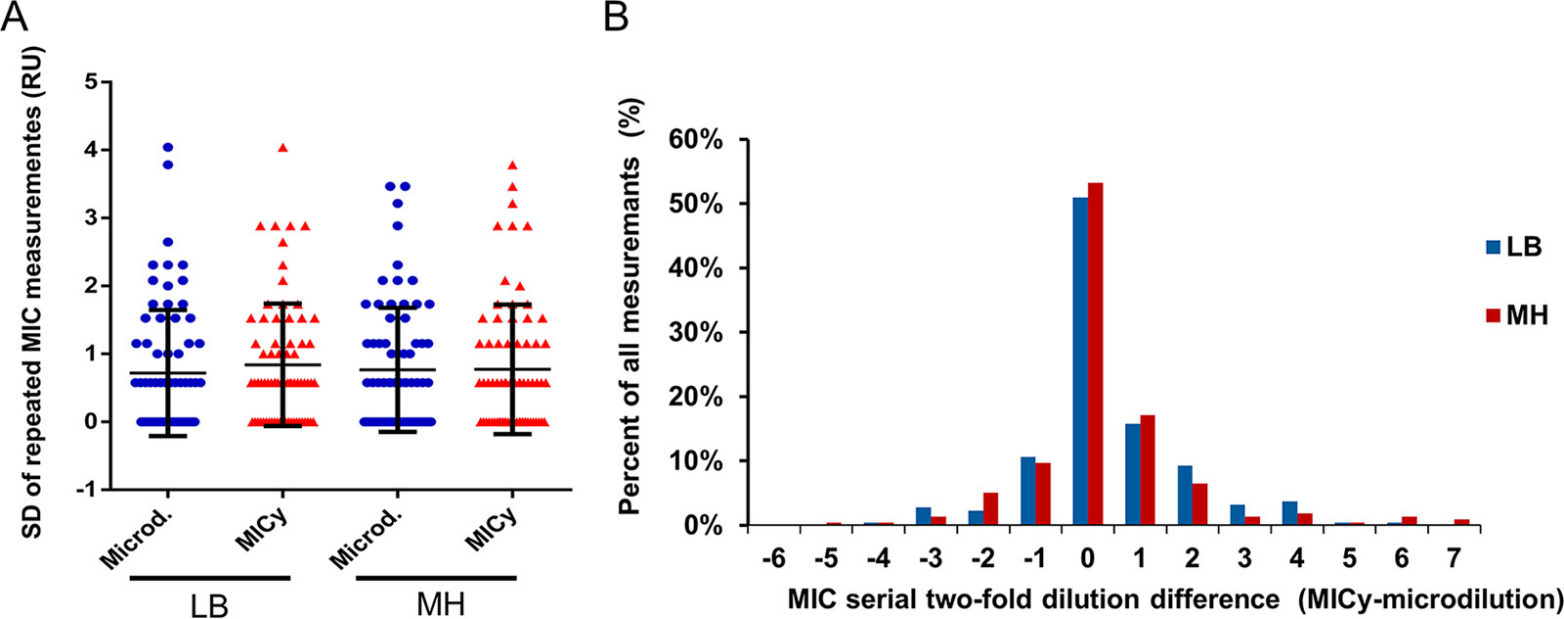
(A) Comparison of reproducibility of MICy and microdilution. Relative units of *y* axis represent SD of 3 independent replicates, where “1” represents a 2-fold dilution difference. (B) Distribution of MIC differences of the two tested methods. Negative values represent lower MIC defined by MICy, positive values represent higher MIC defined by MICy, *n* = 216 both for LB (blue bars) and MH (red bars).

Next, we compared the MIC values generated by the two methods (Table S2). Fig. S2B shows the MIC value comparison of E. coli measurements originating from three experiments carried out independently on three different days. Since this example of E. coli measurements shows that replicates scatter in both methods, we compared standard deviations (SDs). The pattern of the SDs and the average SD of MICy were similar to the control method (Fig. 2A). This suggests that the reproducibility of MICy is comparable to the microdilution.

Similar to the previous point-by-point data comparison, we found a good correlation between MIC values. The overall MIC essential agreement (MIC pairs matched within ±1 dilution range) in MH was over 80% and the weighted Fleiss’ kappa showed a substantial inter-observer agreement (Table 2). We performed discrepancy resolution testing according to the new ISO/DIS 20776-2 (2021) standard (www.isa.org; downloaded on 10/10/2021) to reveal the true rate of essential agreement (EA). This resulted in over 94% EA in Gram negative and over 83% EA in Gram positive bacteria. We also investigated the bias of MICy. According to the data of the point-by-point comparison, the MICs defined by MICy were slightly higher than the MICs from microdilution (Fig. 2B). We calculated the bias of MICy measurements according to the ISO/DIS 20776-2 standard. The percentage of MICy results greater than the reference method was 39.9% (71 measurements from 178), and the percentage of MICy less than the reference was 22.7%, thus the calculated bias was 17.2%.

**TABLE 2:** Comparison table of MIC values originating of microdilution and MICy^*a*^

Broth	Bacteria	Measurement	Essential agreement	Discrepancy resolution testing EA	Weighted Fleiss’ kappa ± SE
Both	All	432	340 (78.7%)	83.3%	0.714
LB	Gram positive	108	88 (81.5%)	69.4%	0.66
	Gram negative	108	79 (73.1%)	86.1%	0.731
LB sum	Both	216	167 (77.3%)	77.8%	0.706
MH	Gram positive	108	83 (76.9%)	83.3%	0.662
	Gram negative	108	90 (83.3%)	94.4%	0.76
MH sum	Both	216	173 (80.1%)	88.9%	0.72

a Essential agreement represents the number and percent of MIC values originating from MICy that were in ±1 2-fold dilution range to the reference method. Essential agreement percent of discrepancy resolution testing was calculated according to ISO/DIS 20776-2:2021 standard.

### Analysis of susceptibility profiles.

**FIG 3:**
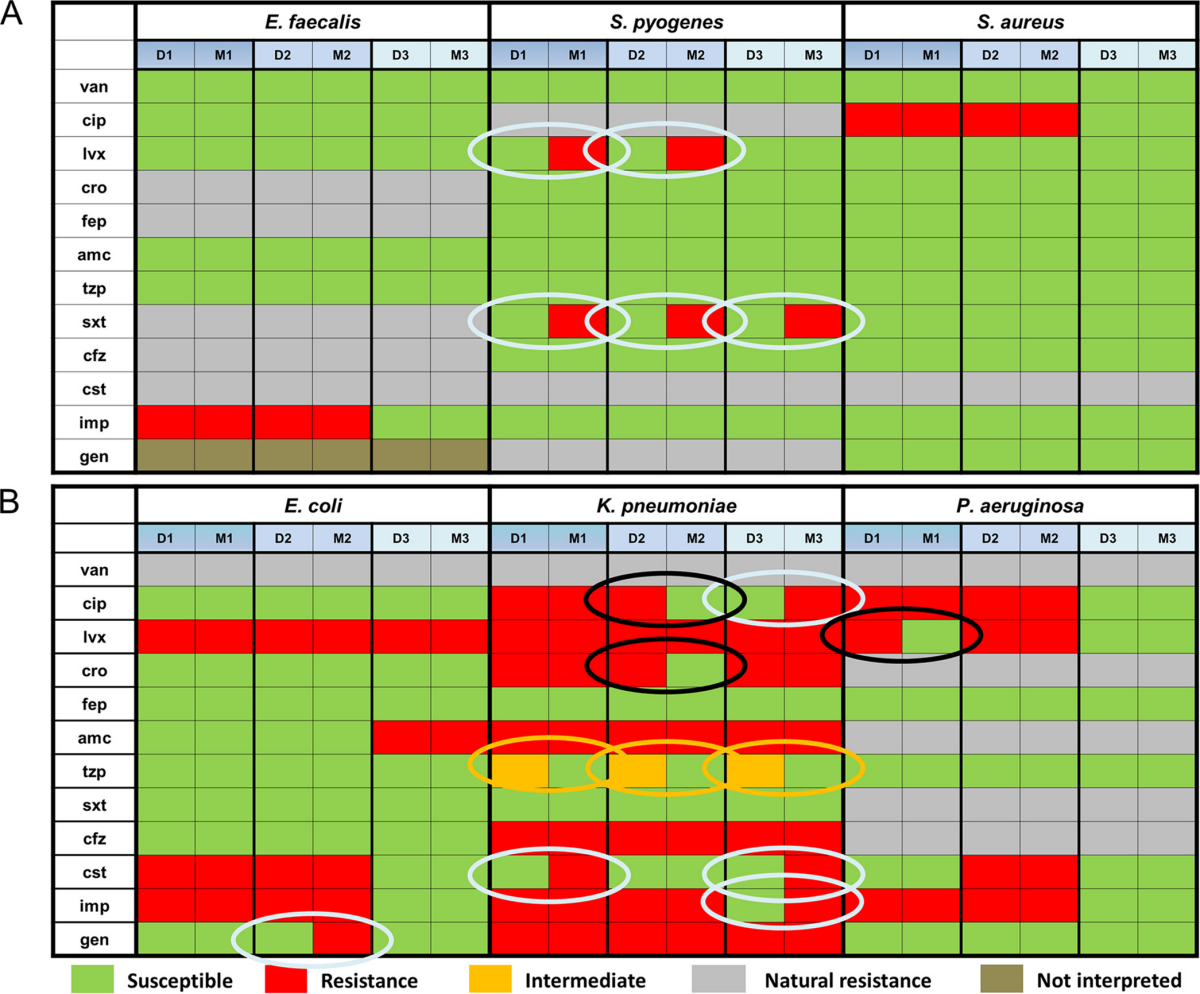
Comparison of susceptibility profiles generated by microdilution and MICy. (A) Gram positive bacteria, (B) Gram negative bacteria. Microdilution (“D”) and MICy (“M”) measurements are paired. Arabic numbers indicate independent replicates. Green box represents susceptibility, red represents resistance, gray represents natural resistance, orange represents intermediate susceptibility, and brown indicates bacteria antibiotics combinations that were not interpreted. Orange circles show minor errors, light blue circles show major errors, black circles show very major errors. *n* = 216.

Susceptibility profiles were generated by comparing measured MIC values in MH to the EUCAST database (Version 9.0, 2019.) breakpoints (Fig. 3). The non-interpretable antibiotics and bacteria combinations were not analyzed any further. In case of Gram positive bacteria there was only a 7.7% major discrepancy between susceptibility results without any minor or very major error (resistant in microdilution but sensitive in MICy) (Table 3). Worse correlation was seen in the profile of Gram negative bacteria. MICy and microdilution resulted in 92% identical decisions. The rate of major discrepancy was 8.7%, and it was 3.2% for very major discrepancies. Intriguingly, most of the inter- and intra-test discrepancies were found in the case of ESBL producing K. pneumoniae. It should be noted that MICy showed resistance in all combinations where natural resistance is known. The summarized susceptibility agreement resulted in a kappa value over 0.84 in Fleiss’ inter-observer test that was referred as *almost perfect* agreement according to Landis (28); however, the rate of major and very major discrepancies was above the limits demanded by the standards.

**TABLE 3:** Summary table of comparison of susceptibility profiles defined by microdilution or MICy^*a*^

Bacteria	Measurement	R/R	S/S	Match %	Minor discrep.	Major discrep.	Very major discrep.	Fleiss’ kappa ± SE
Gram positive	105	40	60	95.2%	0 (0%)	5 (7.7%)	0 (0%)	0.865 ± 0.049
Gram negative	108	52	45	89.8%	3 (2.8%)	5 (10%)	3 (5.5%)	0.795 ± 0.058
Both	213	92	105	92.5%	3 (1.4%)	10 (8.7%)	3 (3.2%)	0.849 ± 0.036

a “R” represents resistance “S” represents susceptibility. Minor, major, and very major discrepancies were calculated according to ISO 20776-2 (2007) standard.

## DISCUSSION

Fast microbiological diagnostics, especially pathogen identification and AST, are important steps for appropriate clinical decision-making, a must for successful and cost-effective treatment of infectious diseases. In this work, we present a simple FC based AST method that produces susceptibility profile with low failure rate a workday earlier than the microdilution method.

**FIG 4:**
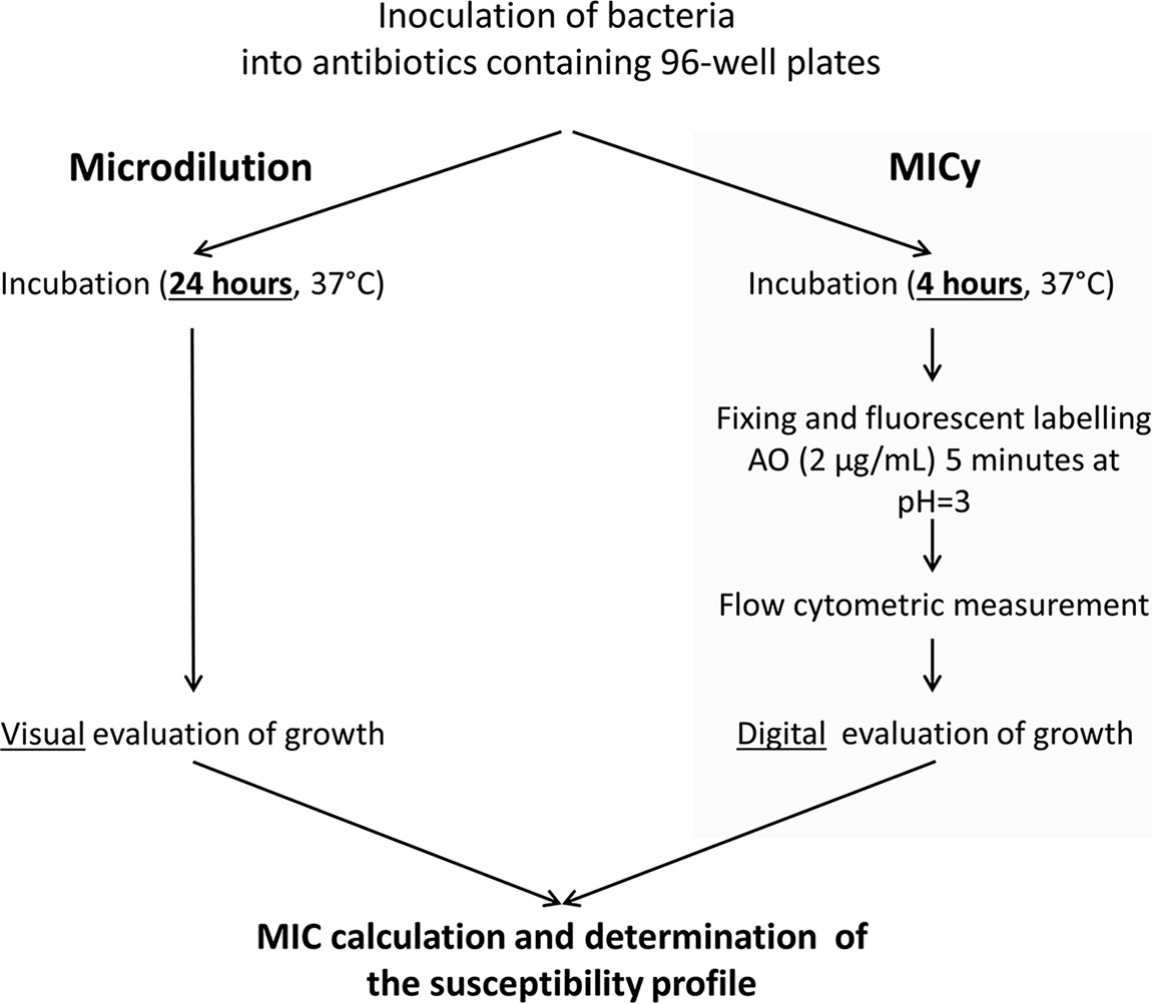
Experimental workflow of microdilution and MICy.

The experimental setup of MICy is based on the microdilution (Fig. 4), but the result can be read after 4 h of incubation time. The time advantage of MICy comes from the more sensitive detection of bacterial count changes by FC compared to visual inspection. We also demonstrated that stopping the incubation and fluorescent labeling can be performed as a one-step process. We used AO fluorescent dye to stain bacteria that was intensive enough to be used under common laboratory circumstances (Fig. 1A and B). AO labels bacteria irrespective of their viability as it binds the nucleic acid content of both living and dead cells (29). This attribute makes our MICy method similar to turbidity-based classical methods. Other advantages of AO are the low costs (for 1 million data points it costs circa $150) and the simple fluorescent excitation, which can be important aspects for later clinical usage.

Speeding up the susceptibility testing is beneficial; however, inadequately short incubation time can result in misleading susceptibility profile and may lead to an inappropriate clinical decision. Earlier studies reported 90 min or even shorter testing time for FC based AST (22, 23, 26). In our hands, a minimum of 4 h incubation time was needed for reliable detection of bacterial growth (Fig. 1C and D). For good quality phenotypic AST, the incubation time should be long enough to allow logarithmic bacterial growth of slower multiplying species as well. Too short an incubation period can deteriorate the quality of results, and on the other hand, longer incubations reduce the time advantage of a test. The limitation of the present study is that all examined bacteria were ATCC isolates with relative fast duplication cycle, but isolates from clinical samples may grow slower, thus longer incubation time could be needed for clinical tests. The optimization of our approach, therefore, should be performed also with these strains. However, in the case of slower growing bacteria, the time advantage of MICy may be more explicit compared to methods based on turbidity changes.

We compared the reproducibility and reliability of MICy to the gold standard method. According to the SD of replicates, the reproducibility of MICy did not significantly differ from the microdilution (Fig. 2A). The overall point-by-point inter-observer agreement between MICy and the gold standard method was over 87%, and importantly, no discrepancy was observed in case of intrinsically resistant bacteria and antibiotics combinations (Table 1 and Table S1 and 2). Based on this, 80% of the calculated MIC pairs fit together within one dilution range in MH. Moreover, essential agreement ratio was above 88% by calculating the discrepancy resolution test according to the ISO/DIS 20776-2:2021 standard (Table 2), and the bias of MICy (17.2%) was in the range required by the standard (≤±30%). Finally, the discrepancy rates of the susceptibility profiles of the MICy were 8.7% for major discrepancies and 3.2% for very major discrepancies (Table 3). Although these results do not fully meet the criteria required by international standards (EA ≥ 90%, bias ≤±30%, major and very major discrepancy rates ≤3%), MICy’s achievement is a promising basis for further investigations to refine the methodology in order to fit in the criteria.

Beyond the time and quality performance, other aspects of antibiotic susceptibility testing were investigated. An ideal AST should be capable of high throughput and automatized processing and produce minimal amount of contaminated waste (10). FC is a robust technique and the technical improvement of the FCs ensured its leading position in high-throughput measurements. The simple data processing—MICy measures bacterial count and calculates the MIC values for 12 antibiotics in circa 60 min—makes it possible to automatize the test. Moreover, the defined phenotypic MIC values can be interpreted according to the clinical breakpoint standards of EUCAST. Thus, by fitting MICy to existing microbiological experience, there may be no need to generate new clinical standards. The costs of the consumables and the waste production of a single MICy test are comparable to the microdilution method; the extra materials used for the sample preparation before FC measurements were the followings: 0.5 ml HBSS/data points, HCl to adjust pH of HBSS to 3, 1 μg Acridine orange/data points (1 g AO costs circa $150), one FC tube/data point, and a few pipet tips for pipetting samples from plates into FC tubes. These costs could be reduced further with an FC that measures directly from a 96-well plate. The only significant extra need of MICy testing is the FC device and its regular maintenance. However, regarding the life span of a modern FC, instrumental costs are minimal per one MICy, especially when compared to the expected advantages of earlier adequate antimicrobial treatment.

In conclusion, we present a simple method for rapid susceptibility testing based on flow cytometry that may have great diagnostic potential. To reveal the real time advantage and the clinical applicability of MICy further testing is needed on clinical samples that can harbor a range of resistance mechanisms such as ESBL production.

## MATERIALS AND METHODS

Hanks' Balanced Salt Solution (HBSS) was from GE Healthcare (South Logan, UT, USA). The Mueller-Hinton broth (MH) and the ingredients of Luria–Bertani broth (LB) were from Sigma-Aldrich (St. Louis, USA). Acridine orange (AO, N,N,N′,N′-Tetramethylacridine-3,6-diamine) was from Serva-Feinbiochemica (Heidelberg, Germany). Fixing solution was prepared from HBSS by adding AO to reach a final concentration of 2 μg/ml and HCL to adjust pH to 3. The 96-well polystyrene plates were from Tomtec (Budapest, Hungary). Antibiotics (vancomycin, ciprofloxacin, levofloxacin, ceftriaxone, cefepime, amoxicillin/clavulanate, piperacillin-tazobactam, trimethoprim-sulfamethoxazole, cefazolin, colistin, imipenem, gentamicin) and all other reagents were of research grade. Escherichia coli (ATCC:25922), Klebsiella pneumoniae (ATCC:700603), Pseudomonas aeruginosa (ATCC:27855), Enterococcus faecalis (ATCC:29212), Streptococcus pyogenes (HNCMB 80003) and Staphylococcus aureus (ATCC:29213) were used as test bacteria.

### FC detection of bacteria.

FC measurements were carried out in a BD FACSCalibur (Franklin Lakes, USA). Since the size of most bacteria is between 500 and 1000 nm, a conventional FC is able to detect and count bacteria as single particles (20, 26, 30). However, the size of smaller bacteria is near to the detection limit (ca. 300 nm) of a conventional FC (31). Therefore, to improve the detection reliability, fluorescent labeling was used to count bacteria (Fig. S1). Fixing solution was used for setting the thresholds to eliminate instrumental noise detected by the side scatter (SSC) and the “green” fluorescence detector (530/30 nm) (Fig. S1A). The upper size limit of bacterial detection was set with 3.8 μm fluorescent beads (SPHERO Rainbow Alignment Particles from Spherotech, USA). The lower SSC threshold was set to exclude 90% of the instrumental noise. Bacteria were enumerated in the R1 gate. The optimal flow rate was defined with a 10-fold serial dilution scale of fluorescent bacteria to avoid swarm detection (27, 30)—it was held under 2000 events/s. FC data were analyzed with Flowing 2.5 Software (Turku Centre for Biotechnology, Finland). Fig. S1 shows representative dot plots of E. coli at the start (C) and the end (D) of a 4 h incubation in MH.

### Determination of optimal incubation time for flow cytometric AST.

To determine the shortest incubation time to detect bacterial growth and to control the linearity of FC measurements, we inoculated 90 μl MH or LB medium with bacteria (10 μl from 10-fold diluted 0.5 McFarland standard) and monitored their growth in three independent experiments. Samples were taken every hour up to 6 h into 500 μl fixing solution. The final pH of the fixing solution with the added sample was pH 3. After fixing the samples for 5 min, they were measured by FC as described above.

### Antibiotics layout for AST.

The layout of antibiotics was the same for both microdilution and MICy for all six tested bacteria irrespective of their natural resistance or sensitivity (Table S1). All antibiotics were applied in standard polystyrene plastic plates in seven different concentrations as described in Table S1. In each plate there were four parallel wells for positive control that contained broth without antibiotics inoculated at the beginning of incubation, and four wells for negative control that were not inoculated at all. To measure the initial bacterial count, four wells were inoculated after the incubation period immediately before the fixing with the same amount of bacteria (that were stored at 4°C in saline).

### MIC determination.

Quantitative antibiotic susceptibility levels of bacteria were measured by determining MIC values according to the guidelines of EUCAST. The MIC originating from MICy was compared to the gold standard method. Microdilution data were analyzed after 24 h (due daily work schedule issues), a deviation from the standard 16–20 h period (Fig. 4). In case of MICy, bacteria were transferred into sterile 0.9% NaCl solution to reach solution turbidity equivalent to a 0.5 McFarland standard. Bacteria were further diluted 10-fold with saline. A 96-well plate with the described antibiotics layout (Table S1) were inoculated similar to the microdilution method. Wells contained 90 μl broth that were inoculated with 10 μl bacterial solution. The plates were sealed and incubated at 37°C under aerophilic conditions. At the end of the 4 h incubation, 90 μl of inoculated broth was added to 500 μl fixing solution. After 5 min fixing, samples were measured by FC.

### Statistics.

Statistical analyses were performed with the on-line version of GraphPad Prism (https://www.graphpad.com/quickcalcs/ accessed on January 7, 2020, La Jolla, CA, USA). Fleiss’ Kappa was used to assess the agreement between microdilution and MICy. McNemar test was used to analyze the bias between microdilution and MICy. One-way RM ANOVA analysis was performed using GraphPad Prism 6 (La Jolla, CA, USA). Difference was taken significant if the *P* value was <0.05.
