# Supplementary material for: MICy: a Novel Flow Cytometric Method for Rapid Determination of Minimal Inhibitory Concentration
Source: Microbiol Spectr. 2021 Dec 8;9(3):e00901-21. doi: 10.1128/spectrum.00901-21 (PMC8653823; doi:10.1128/spectrum.00901-21)
Supplement: SUPPLEMENTAL FILE 3 — Supplemental material. Download SPECTRUM00901-21_Supp_1_seq8.pdf, PDF file, 0.7 MB [file spectrum00901-21_supp_1_seq8.pdf]

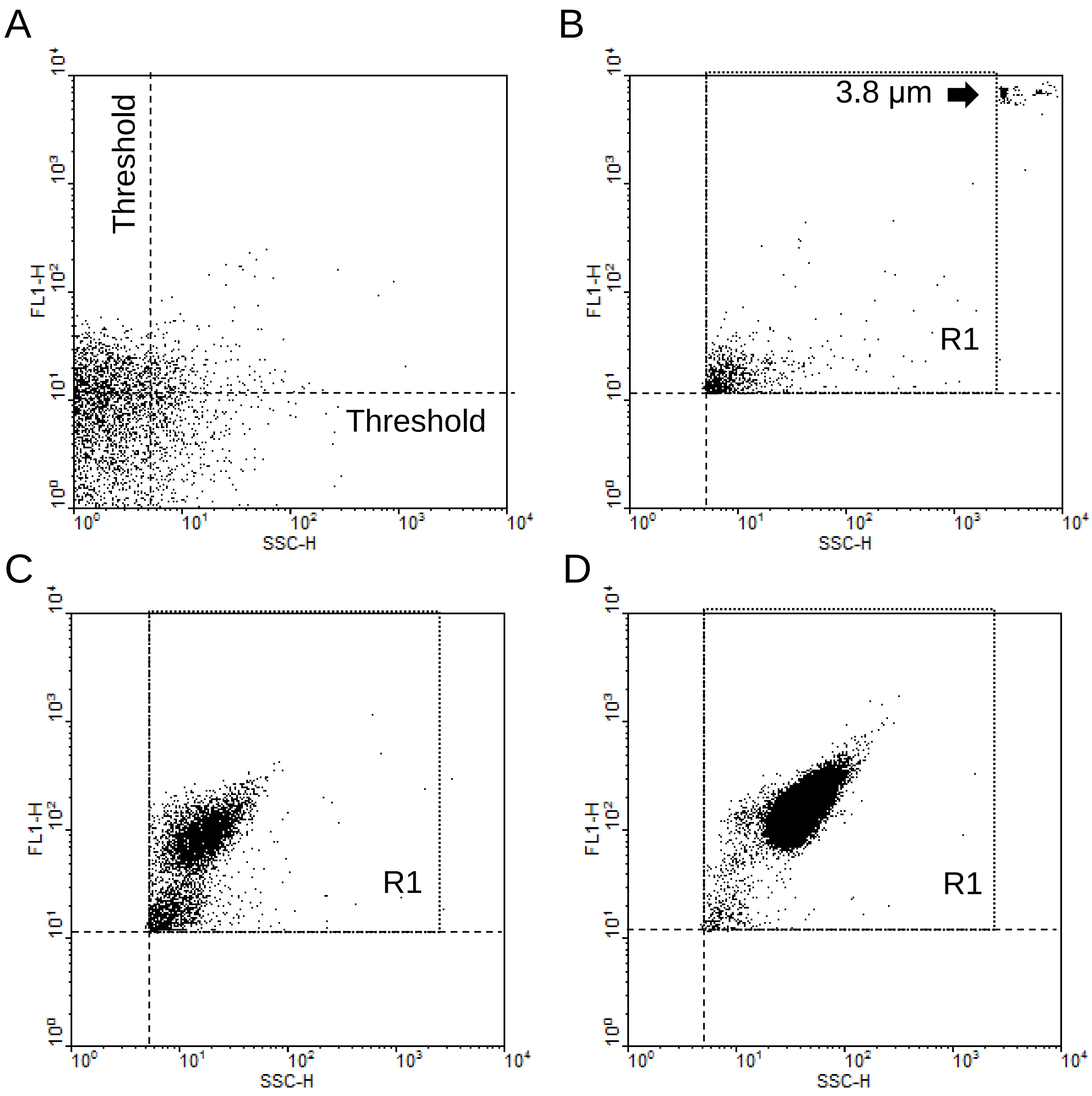

Suppl. Fig. 1.

Supplementary Figure. 1 A *Gating strategy of FC detection of bacteria*. Representative dot plots of FC measurements. **A** Fluorescence and SSC thresholds were set using the fixing solution to exclude instrumental noise. **B.** Spherotech beads (3.8µm) were used to set the upper size limit of bacterium detection gate (R1); lower size limit was set to the SSC threshold. **C.** Representative dot plot of AO labelled *E.coli* inoculated into MH broth. **D**. Representative dot plot of AO labelled *E.coli* after 4 hours incubation in MH broth.

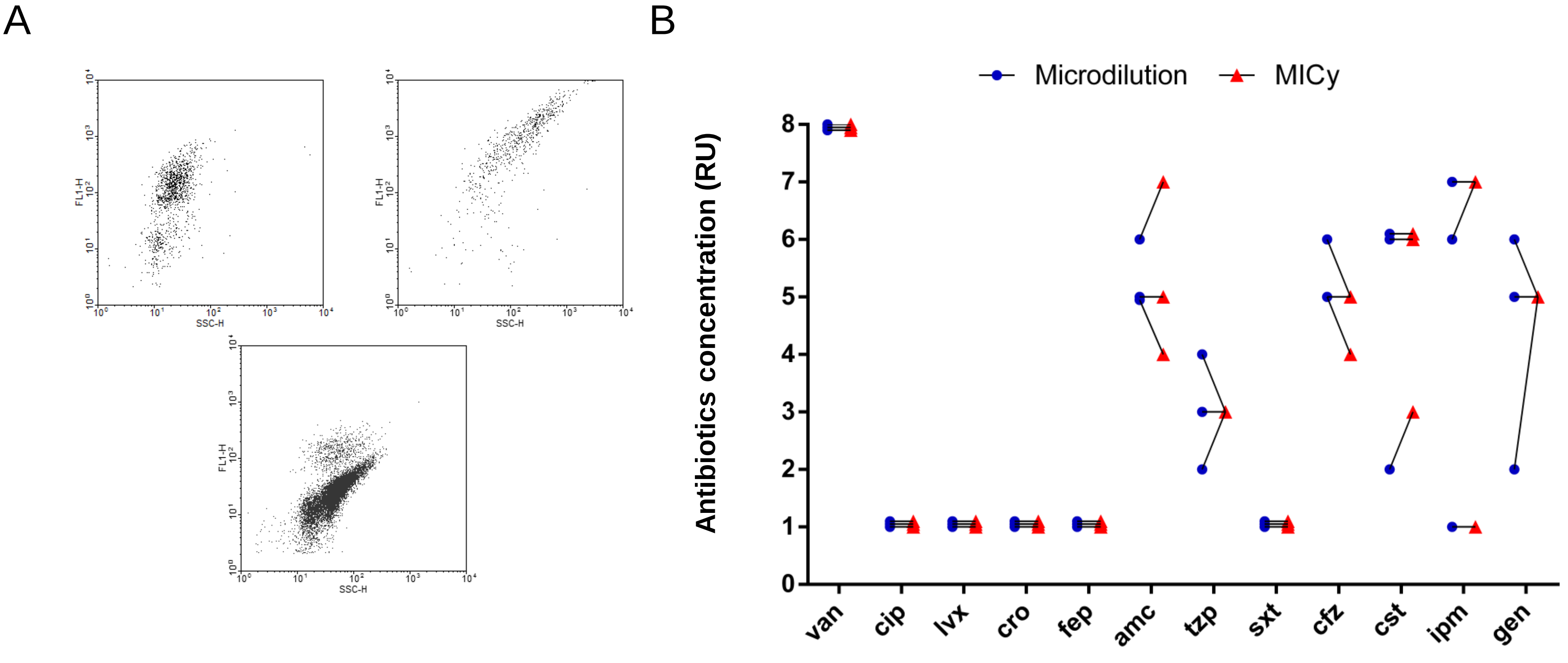

Suppl. Fig. 2.

Supplementary Figure 2. A FC presentation of *S. aureus* aggregation due to antibiotic effect. Representative dot plots. *S. aureus* incubated with 8 µg/ml (upper left), 1 µg/ml (upper right) and 0.25 µg/ml (lower left) levofloxacin. **B** Comparison of MICs originating from MICy (red triangles) to the results of microdilution (blue dots) in case of three independent replicates of *E. coli* AST in MH. Black line connects the MIC pairs. Relative units of y-axis represent two-fold serial dilutions of antibiotics: value '1' represents inhibition at the lowest tested antibiotic concentration, '8' represents growth at the highest concentration.
